# The Contribution of Bullying Involvement and Alexithymia to Somatic Complaints in Preadolescents

**DOI:** 10.3390/children10050905

**Published:** 2023-05-21

**Authors:** Valentina Levantini, Marina Camodeca, Nicolò Maria Iannello

**Affiliations:** 1 IRCCS Stella Maris, Scientific Institute of Child Neurology and Psychiatry, 56128 Pisa, Italy; 2 Department of Languages and Literatures, Communication, Education, and Society, University of Udine, 33100 Udine, Italy; 3 Department of Law, University of Palermo, 90134 Palermo, Italy

**Keywords:** bullying, victimization, outsider, somatic complaints, middle school, affect/emotions

## Abstract

Somatic complaints during preadolescence are connected to individual and contextual factors, and extant research highlights the relevance of alexithymia and bullying involvement. In this cross-sectional study, we explored the joint and unique influence of bullying involvement—as perpetrators, victims, or outsiders—and alexithymia on somatic complaints in a sample of 179 Italian middle-school students (aged 11–15). Findings revealed an indirect association between bullying perpetration and victimization complaints through alexithymia. We also found a significant direct association between victimization and somatic complaints. No significant association between outsider behavior and somatization was found. Our results revealed that bullying perpetration and victimization could increase youths’ risk for somatic complaints and clarify one of the processes underlying this association. The current findings further emphasize the relevance of emotional awareness for youths’ well-being and propose that implementing social–emotional skills might prevent some of the adverse consequences of being involved in bullying episodes.

## 1. Introduction

Many children and adolescents suffer from physical pains (e.g., headache and stomachache), which can occur in chronic and severe forms or as a combination of multiple sorts of pain [1]. Although a medical cause can sometimes be found, an organic etiology can explain somatic complaints in only a few cases, whereas sociopsychological factors can play a significant role. Noteworthily, adolescence and preadolescence are sensitive periods for the onset of somatic complaints [2] because youths have to deal with several developmental challenges [3]. In particular, during preadolescence, individuals go through a number of changes occurring at different levels [4]. For instance, rapid bodily modifications characterize this stage of life [5]; concurrently, preadolescents are exposed to an expanding social environment and have to adjust to new school settings and relationships [6]. In addition, less positive emotions and emotional swings are more frequently experienced [4,7]. Arguably, all these individual and social transformations might act as stressors making youths vulnerable to somatic complaints, which, due to organic or nonorganic causes, interfere with young people’s quality of life and are frequently associated with psychological maladjustment [8,9].

### 1.1. Involvement in Bullying

The development of somatic complaints has been linked to both contextual (e.g., distressing life events) and individual (e.g., personality traits) factors [8]. Among the contextual aspects, school bullying is a potential risk for somatic problems, especially for youths (for a review, see [10]). Bullying refers to the systematic abuse of power in which proactive, unjustified, and frequent acts of aggression are perpetrated toward peers considered weaker or less powerful [11,12]. Although not directly involved as bullies or victims, the literature claims that almost all classmates are present when a bullying attack occurs or are at least aware of it [13]. Whereas some support the bully and others defend the victim, many prefer not to take sides and shy away; these youths have been labeled outsiders [14]. Enacting aggression, being the target of harassment, or being an outsider can be considered highly distressing experiences that lead to long-lasting behavioral, social, and psychological problems [15,16]. Notably, some studies found that both bullies and victims are more at risk of suffering from somatic complaints than their uninvolved peers [17,18], although victims present the highest incidence [19]. Consistently, a longitudinal study found that being victimized at the beginning of the school year was a predisposing condition for developing somatic complaints across time [20]. Additionally, the literature reports that even witnessing others being harassed or maltreated can be highly distressing and lead to adverse outcomes, such as increased anxiety, empathic distress, and mental health risks across several indicators, including somatization [21,22,23,24]. Witnessing peers being bullied without taking action can be a disconcerting experience, often followed by considerable negative affects, to the point that outsiders have been sometimes considered “co-victims” of bullying (e.g., [25]). Despite the wealth of studies showing an association between involvement in bullying and somatic complaints, little is known about the mechanisms that can account for it. Broadening our knowledge about the factors that might link bullying involvement to adverse outcomes would also further our insights into the causes of such common conditions and their prevention.

### 1.2. The Role of Alexithymia

Regarding the individual factors that might foster the development of somatic complaints, studies have highly emphasized the role of alexithymia (and its related construct of emotion awareness) as one of the most significant predictors of such conditions in adult populations [26]. Similar results have been found in studies with children and adolescents (e.g., [27,28]), and a recent systematic review [29] revealed that seven out of the eight studies comparing children with medically unexplained symptoms and healthy controls found that the former had higher levels of self-reported alexithymia.

Alexithymia refers to the difficulty in identifying, describing, and differentiating one’s emotions and distinguishing feelings from bodily sensations [26]. Alexithymic individuals can neither relate their affective conditions to specific situations nor react to events with appropriate emotional responses [28,30]. It has been proposed that alexithymic individuals might have difficulties in mentally representing affective arousal [31], which would interfere with youths’ ability to reason on their feelings, relate these to the eliciting events, and ultimately regulate their emotions [32]. Due to the inability to mentalize the bodily sensations associated with affective arousal and to analyze the situation from an external point of view, adolescents are not able to use their emotions to understand themselves and the cause of their pain. This would eventually lead to a greater focus on the signs of emotional arousal (e.g., fast heartbeat), eliciting further anxiety and overwhelming distress, which is more likely to be misinterpreted as sickness and an index of body malfunction [27,33]. Lumley et al. [34] explained that alexithymia might lead to somatic complaints because it gives rise to undifferentiated arousal, which produces somatic sensations; these sensations, in turn, are amplified by alexithymic individuals who attribute them to illness-related causes rather than to psychological ones.

Even though the literature about the influence of alexithymia on youths’ somatic complaints cannot be considered conclusive, given the scant level of evidence currently available, it surely highlights the relevance of this construct, calling for more research. Furthermore, alexithymia might also assume particular relevance in the context of bullying involvement, which represents a highly distressing and drastically common experience for youths, especially during the middle-school years [35,36]. Indeed, although alexithymia is usually considered a dispositional characteristic [37], it might be intensified by adverse experiences and develop as a consequence of traumatic or highly distressing events, and it might mediate the association between these experiences and different negative outcomes [38,39,40]. According to this perspective, authors who consider alexithymia as a state influenced by stressful situations or external prompts suggest that alexithymia can be interpreted as a defensive mechanism to face those events and deal with the negative emotions elicited by them. In this regard, some authors have proposed the existence of a form of reactive alexithymia that “functions as a state reaction to mitigate painful affects” [41] (p. 284), also called “secondary alexithymia” [41].

Along this line, only a few studies investigated the relationship between alexithymia and bullying involvement, and they primarily focused on victimization. However, these studies consistently reported an association between the two variables, with alexithymia mediating the link between victimization and severe outcomes, including post-traumatic stress symptoms [42], self-harm attempts [43], and internalizing and externalizing problems [44] among adolescents and preadolescents. Even though less evidence is available on this matter, some studies also reported an association between bullying and higher levels of alexithymia. For instance, Wachs and Wright [45] found, in a large sample of youths aged 12–18 years, that bullies scored higher in alexithymia than non-bullies, and that these scores were higher for those who were both traditional and cyberbullies than for those who bullied in only one modality (see also [46,47]). To the best of our knowledge, no previous study investigated the link between outsider behavior and alexithymia.

### 1.3. The Current Study

The literature on somatization has usually selectively focused either on individual characteristics [8,48] or aversive/distressing life events [49]. However, it has been argued that a promising way to explore the onset of somatic complaints among teenagers would be concurrently taking into account adolescents’ intrapersonal factors and their environment [50]. Indeed, an ecological framework [51] would provide a more nuanced picture of those individual and contextual variables that may expose youths to somatization. In line with this perspective, the present cross-sectional work investigates the joint and unique contribution of bullying involvement (contextual factor) and alexithymia (intrapersonal factors) to somatic complaints in a sample of Italian middle-school students. As bullying perpetration and victimization peak during middle-school years [35,36], it seemed relevant to better understand both their associations with somatic complaints and the mechanisms underlying such relations among preadolescents.

We propose that perpetrating harassment and suffering from or witnessing bullying might represent highly distressing events able to impair youths’ emotional functioning and well-being. Adolescents involved in bullying may develop somatic complaints due to difficulties in processing emotions, which, by increasing their arousal and hindering their capacity to analyze internal sensations properly, might make them prone to express this distress through bodily symptoms. On the basis of the literature suggesting that alexithymia is associated with both bullying involvement and somatic complaints [24,45] and might mediate the link between victimization and severe outcomes [42,43], we tested whether alexithymia mediates the link between victimization and somatic complaints. We then further explored whether this mechanism might also shed light on the association between bullying or outsider behavior and somatization reported by the extant literature (e.g., [19,23]).

Lastly, the literature suggests that boys, compared with girls, are more often involved in bullying as perpetrators [52], whereas girls usually report higher levels of both alexithymia and somatic complaints than boys [53,54]. On this basis, we controlled for gender in the analyses.

## 2. Materials and Methods

### 2.1. Participants and Procedure

The sample included 179 middle-school students (49.20% males) aged 11 to 15 years (*M* = 12.40 years; *SD* = 0.94), attending two different schools located in a big city in Northern Italy. Fifty students (27.90%) were sixth graders, 74 (41.30%) were seventh graders, and the remaining 55 (30.70%) were eighth graders.

Students were asked to complete a series of questionnaires assessing their involvement in bullying as perpetrators, victims, and outsiders, as well as the presence of somatic complaints and alexithymia. Data were collected in the classrooms, during school time, by a trained master student in psychology.

School principals and teachers approved the project and allowed data collection. All parents signed a written informed consent form to let their children participate. The study was conducted according to the guidelines of the Declaration of Helsinki and was approved by the Institutional Review Board of the University of Milano-Bicocca (Milan, Italy; protocol number 237).

### 2.2. Measures

*Somatic Complaints.* Somatic complaints were assessed using the Somatic Complaint List [55], an 11-item self-report questionnaire. Students were asked to rate on a three-point Likert scale (0 = *never*; 1 = *sometimes*; 2 = *often*) the frequency with which they recently experienced different somatic complaints (e.g., stomachache, headache, tiredness, pain, weakness). Reliabilities were calculated as the greater lower bound (glb) index, representing the lowest value of the real reliability and ranging from glb to 1 [56]. In the current sample, glb was 0.87.

*Bullying, Victimization, and Outsider Behavior.* Bullying perpetration, victimization, and outsider behavior were assessed by four items each, covering different forms of bullying (e.g., [57]). Students were asked to report the frequency, during the current school year, with which they had bullied someone (e.g., “I tease and offend some classmates, give them nasty nicknames, or threaten them”), had been victimized (e.g., “I am often excluded or isolated from the group”), or passively witnessed someone being bullied (e.g., “When a classmate is hit or pushed, I stand by and I mind my own business”). The response format was on a four-point scale (from *never* to *almost always*). In the current sample, glb was 0.52 for bullying, 0.78 for victimization, and 0.71 for outsider behavior. Since these values can be affected by short item scale length, we also explored the mean interitem correlations (MICs) for the self-report scores for bullying (MIC = 0.19), victimization (MIC = 0.38), and outsider behavior (MIC = 0.36), whose optimal values range between 0.15 and 0.50 [58]. Therefore, even though the reliability for bullying was low, the mean interitem correlations (MICs) showed that the items in the scale were sufficiently correlated and measured the same construct.

*Alexithymia*. Alexithymia was evaluated using the Italian version of the Alexithymia Questionnaire for Children [28,59]. The questionnaire consists of 20 items, evaluating three aspects of alexithymia: difficulty in identifying feelings (e.g., “When I am upset, I do not know if I am sad, scared or angry”), difficulty in describing feelings (e.g., “I find it difficult to say how I feel inside”), and externally oriented thinking (e.g., “I prefer talking to people about everyday things, rather than about how they feel”). Students were instructed to score each item on a three-point response scale (0 = *not true*; 1 = *a bit true*; 2 = *true*). For the purpose of this study, a total score was obtained by summing all the items. In the current sample, glb was 0.87.

### 2.3. Statistical Analyses

**Figure 1:**
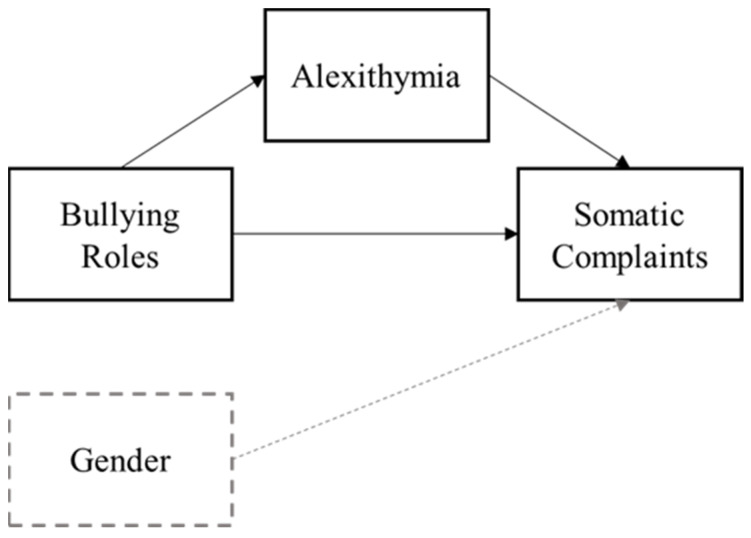
Theoretical mediation model of the association between bullying involvement and somatic complaints via alexithymia.

All statistical tests were run on IBM SPSS Statistics for Windows, Version 26.0. As preliminary analyses, we ran *t*-tests to look at the differences in the study variables between the two schools. Then, we computed descriptive statistics and bivariate correlations among the main study variables, as well as between them and gender. To test the mediating role of alexithymia, we ran three regressions using Model 4 in the PROCESS v.4.0 macro for SPSS [60]. Bullying, victimization, and outsider behavior were used as independent variables, somatic complaints were used as the dependent variable, alexithymia was used as the mediator, and gender was used as a covariate (see Figure 1). Both the direct and indirect effects of the independent variables on somatic complaints were tested. The indirect effects were interpreted using 95% bootstrap confidence intervals (BootCIs).

## 3. Results

The *t*-tests showed that there were no significant differences between the two schools involved in the study regarding somatic complaints (*t* = −1.386, *p* = 0.167), alexithymia (*t* = −1.048, *p* = 0.296), and involvement in bullying (bullying: *t* = −0.142, *p* = 0.887; victimization: *t* = −0.0686, *p* = 0.494; outsider behavior: *t* = 0.216, *p* = 0.829)

Table 1 displays the descriptive statistics and the zero-order correlations among the study variables. The results showed that gender, bullying, and victimization were significantly and positively correlated with alexithymia and somatic complaints. The results also showed a significant and positive association between alexithymia and somatic complaints. Outsider behavior did not correlate with any variable.

**Figure 2:**
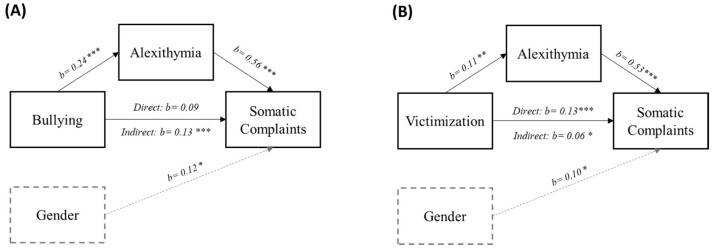
Direct and indirect effects of bullying (**A**) and victimization (**B**) on somatic complaints via alexithymia. Note: b = unstandardized coefficient. Gender was coded as male = 0 and female = 1. * *p* ≤ 0.05; ** *p* ≤ 0.01; *** *p* ≤ 0.001.

As indicated in Figure 2, the regression models showed that being a girl, victimization, and alexithymia were significantly associated with somatic complaints; bullying and victimization were also associated with alexithymia. The mediation analyses showed that alexithymia mediated the relationship between bullying and somatic complaints and between victimization and somatic complaints. Specifically, the findings showed a significant indirect effect of bullying on somatic complaints via alexithymia (*b* = 0.13, 95%BootCIs: 0.621, 0.213). The direct effect of bullying perpetration on somatic complaints was not significant (*b* = 0.09, *p* = 0.202), suggesting a complete mediation (Figure 2A).

Results also showed a significant indirect effect of victimization on somatic complaints through alexithymia (*b* = 0.06, 95%BootCIs: 0.015, 0.107). The direct effect of victimization on somatic complaints was significant (*b* = 0.13, *p* = 0.001), providing evidence for partial mediation (Figure 2B).

Outsider behavior scores were not directly (*b* = 0.03, *p* = 0.485) or indirectly (*b* = 0.03, 95%BootCIs: −0.018, 0.071) associated with somatic complaints or alexithymia (*b* = 0.05, *p* = 0.22).

## 4. Discussion

Being involved in bullying episodes at school as perpetrators, victims, or outsiders is a highly distressing experience that can be related to adverse mental health outcomes, including somatic complaints [17,18,24]. In addition to this contextual variable, individual factors, such as alexithymia, have been associated with somatic problems [10,27]. We reckon that a better understanding of the mechanisms underpinning these associations could yield theoretical and practical advances. The outcomes of the present work, within an ecological framework, preliminarily show the joint and unique contribution of bullying involvement (contextual factor) and alexithymia (individual factor) to somatic complaints.

Bullying perpetration was correlated with somatic complaints, as suggested by some previous studies (e.g., [61]), but this direct association was no longer significant once bullying was modeled with other variables in the mediation analysis. However, we found an indirect link through alexithymia, suggesting a possible mechanism through which bullies may develop somatic complaints. Consistently with previous studies, we found that bullying was associated with higher levels of alexithymia [36], which in turn was associated with more prominent somatization problems. Although their acts cause great suffering to others, bullies themselves usually display poor psychological, emotional, and social adjustment (e.g., social exclusion, externalizing problems, and school dropout [62]), highlighting that violence might also be a challenging experience with significant and long-lasting repercussions for perpetrators. Some studies have suggested that alexithymia can arise in the agents of highly distressing events as a defense mechanism to minimize emotional involvement and deflect negative emotions perceived as threatening [63]. For instance, in people who commit extremely violent behaviors, alexithymia would be related to the regulation and inhibition of the overwhelming emotions associated with these actions [64]. Even though highly speculative, similar mechanisms may intervene in some bullies, who may detach from and suppress the negative and highly activating emotions deriving from persistently harassing and mistreating schoolmates, to protect themselves from the psychological consequences of the violent acts perpetrated. These emotional impairments, which are core features of alexithymia, in turn, are established risk factors for somatic complaints [27,33].

We also found evidence for a partial mediation between victimization and somatic complaints via alexithymia. Indeed, victimization was significantly and directly associated with somatic complaints. This suggests that being victimized by peers is, per se, a risk factor for mental health issues, as also highlighted in some studies exploring the long-term consequences of peer victimization [65]. Moreover, victimization was indirectly linked to a higher frequency of somatic complaints via alexithymia. Alexithymia can increase or develop as a consequence of highly distressing life events [40], and peer victimization surely falls into this category. We can surmise that the persistent and severe bullying experienced might act as an intense stressor, inhibiting any attempt to recognize and label emotions, and leading victims to direct their efforts and thoughts toward the (avoidance of) harassment. In addition, identifying, describing, and analyzing the negative feelings that arise from being bullied (e.g., anger, sadness, rage, and shame) may be too demanding for victims, who prefer to stay away from it and not be overwhelmed by pain. These emotional difficulties, in turn, might make it more likely for victims to interpret the arousal associated with their suffering (e.g., anxiety and depressive symptoms) as illness-dependent symptoms instead of signs of psychological maladjustment. These findings fit within the current literature, showing that alexithymia mediates the association between victimization and several negative outcomes, including internalizing problems [42,43], further emphasizing the importance of emotional awareness for youths’ wellbeing. Various theories and studies have highlighted the relevance of emotional awareness to better understand the risk of youths’ psychological maladjustment [66,67,68]. Adolescence and preadolescence are sensible periods characterized by several changes [4], including hormonal and physical modifications [5] and changes in youths’ social environment (e.g., new school settings and peer relationships) [6]. Moreover, preadolescence and adolescence are also distinguished by a rapid increase in emotional arousal; however, the neural mechanisms underpinning emotional awareness are still developing [3], and youths might not be fully equipped to comprehend, communicate, and manage their emotions appropriately. This might be particularly relevant for youths exposed to highly distressing events, such as victimization, which also tend to increase during middle school [36]. Indeed, exposure to extreme or uncontrollable stressors leads to physiological changes to the stress response systems [69], which might eventually influence the ability to perceive one’s internal bodily states and label them as emotions, increasing victims’ risk for internalizing problems [70].

Lastly, our study did not provide evidence of a direct or indirect link between outsider behavior and somatic complaints, which contrasts with a previous study showing that outsider behavior is associated with somatization problems [24]. Methodological differences might account for this discrepancy. Indeed, unlike Rivers et al. [24], who focused on witnesses in a more general sense, we specifically focused on outsider behavior, here operationalized as the tendency of some individuals to withdraw and not intervene because they believe that bullying is something that does not concern them or in which they do not want to be involved. It is possible that our measure better identifies students who are less emotionally engaged and influenced by witnessing someone being bullied; therefore, they are less at risk of developing emotional or adjustment problems as a consequence. Moreover, outsiders may be a heterogeneous group [71]; thus, these students may differ in the way and degree to which they are affected by witnessing peers being harassed [72]. Accordingly, some of them might head toward more unfavorable pathways while others do not. Future studies should consider a more nuanced operationalization of different outsiders’ attitudes to disentangle the association linking their behavior, alexithymia, and somatic complaints. Lastly, we solely assessed one particular outcome and one mediator, namely, somatic complaints and alexithymia, which might not properly capture the maladjustment experienced by outsiders. Future studies would benefit from evaluating a more comprehensive range of mental health indicators for outsiders.

The results of the current study need to be interpreted in light of some limitations. First, our sample size was small, and we only employed self-report measures, which may be biased by social desirability and lead to shared variance problems. Employing other methods, such as peer nominations for bullying involvement, interviews, or direct observation, may yield different associations among variables. Then, even though participants were assured about the confidentiality of their responses and a collaborator was present to supervise the data collection, we cannot wholly rule out that completing the questionnaires in the classrooms might have slightly influenced students’ responses. In addition, we used a cross-sectional design; thus, we cannot trace alternative explanations for our findings (e.g., reverse or reciprocal relationships between the study variables) or developmental pathways. Therefore, longitudinal studies with ample samples are warranted to corroborate our mediation models and to better understand the relationships among bullying involvement, alexithymia, and somatic complaints. Moreover, we suggest future research to collect sociodemographic information and assess other variables associated with alexithymia and/or somatic complaints (e.g., challenging family context, depressive symptoms, anxiety, empathy, and emotion regulation) [33,43,73]. As previously advanced, our work also highlights the need for more research on the correlates of outsider behavior at school, a currently under-investigated research area with inconsistent results.

Overall, our study confirmed that preadolescents involved as bullies and victims are at risk of developing somatic complaints, and the findings preliminarily shed light on one potential mechanism underpinning this link. The results suggest that addressing emotional awareness and comprehension might help curb some of the effects of bullying involvement. Some school-based anti-bullying prevention programs aim to either identify and target bullies or empower victims and outsiders to effectively reduce bullying perpetration and victimization [74], while others, such as the KiVa and the NoTrap! programs [75,76], also aim to improve social relationships and the school climate. Similarly, interventions addressing students’ mentalization ability aim to create a safer and more cooperative and cohesive environment, as well as improve social relationships, eventually reducing victimization and increasing helpfulness [77]. Despite all these programs being proven effective in reducing school bullying, we believe that the parallel implementation of prevention programs specifically promoting social–emotional skills (i.e., emotional awareness and management) might provide students with valuable tools to cope with the consequences of bullying involvement and, at the same time, promote the establishment of positive relationships between classmates and improve student mental health outcomes. In this regard, a prominent example is the Coping Power Universal (CPU), a socioemotional learning prevention program that has been shown to significantly reduce internalizing problems and increase prosocial behavior in a large sample of middle-school Italian students [78]. The CPU version developed for middle-school students specifically aims to improve their emotional awareness and regulation, recognize and better understand others’ emotions and points of view, and cope with social conflict and peer pressure. This prevention program is implemented by teachers, wholly incorporated into the school routine, and easy to run along with other programs. The combination of these types of prevention programs would, on the one hand, target specific risk factors associated with bullying and, on the other hand, boost those skills that could help students deal with the consequences of stressful experiences.

## Figures and Tables

**Table 1:** Descriptive statistics of study variables and zero-order correlations among them.

		1	2	3	4	5	6
1.	Gender						
2.	Somatic complaints	0.27 **					
3.	Alexithymia	0.19 **	0.54 **				
4.	Bullying	−0.14	0.17 *	0.24 **			
5.	Victimization	0.07	0.33 **	0.23 **	0.10		
6.	Outsider behavior	0.00	0.09	0.11	0.08	−0.04	
	*Mean*	-	0.59	0.81	1.35	1.70	1.84
	*SD*	-	0.35	0.30	0.34	0.59	0.59

Note. *SD* = standard deviation. Scores for somatic complaints, alexithymia, and bullying behaviors were computed as mean scores. Gender was coded as male = 0 and female = 1. * *p* ≤ 0.05; ** *p* ≤ 0.01.

## Data Availability

Data are available from the corresponding author upon reasonable request.
